# Determinant factors of under-five mortality in Southern Nations, Nationalities and People’s region (SNNPR), Ethiopia

**DOI:** 10.1186/s13052-021-01118-0

**Published:** 2021-10-30

**Authors:** Gizachew Gobebo

**Affiliations:** Department of Statistics, College of Natural and Computational Sciences, Ambo University, Ambo, Ethiopia

**Keywords:** Under-five mortality, Determinant factors, SNNPR, Ethiopia

## Abstract

**Background:**

Child mortality is a key indicator of the performance of the health system of a nation. Impressive progress in the reduction of under-five mortality has been made in Ethiopia. However, still there are some regions where the under-five mortality rates are high. Southern Nations, Nationalities, and Peoples’ Region (SNNPR) is among those regions in Ethiopia with high under-five mortality rates. This study aimed to identify the determinant factors of under-five mortality in SNNPR.

**Methods:**

Data used for the study were drawn from the 2016 EDHS. A total of 1277 under-five children were included in the study. A multivariable logistic regression model was fitted to identify determinant factors associated with under-five mortality.

**Results:**

Children with second or third birth order (OR = 1.316, 95% CI: (1.097, 2.343)), fourth or fifth birth order (OR = 1.934, 95% CI: (1.678, 3.822)), sixth or above birth order (OR = 3.980, 95% CI: (2.352, 6.734)) were significantly associated with increased risk of under-five mortality as compared to those with first birth order. Increased risk of under-five mortality was also significantly associated with a family size of five or more (OR = 3.397, 95% CI: (1.702, 6.782)) as compared to the family size of less than five; smaller size at birth (OR = 1.714, 95% CI: (1.120, 2.623)) as compared to larger size at birth; multiple births (OR = 1.472, 95% CI: (1.289, 2.746)) as compared to singletons. On the other hand, female children (OR = 0.552, 95% CI: (0.327, 0.932)), children born at health institutions (OR = 0.449, 95% CI: (0.228, 0.681)) and children who were breastfed (OR = 0.657, 95% CI: (0.393, 0.864)) were significantly associated with decreased risk of under-five mortality as compared to male children, those born at home and those who were not breastfed respectively.

**Conclusions:**

Sex of a child, birth order, size of a child at birth, place of delivery, birth type, breastfeeding status, and family size were significant factors associated with under-five mortality in SNNPR, Ethiopia. Thus, planning and implementing relevant strategies that focus on those identified determinant factors of under-five mortality is required for the improvement of child survival in SNNPR, Ethiopia.

## Background

Child mortality is a key indicator of the performance of the health system of a nation [1] Remarkable progress in the reduction of mortality among children including children under the age of five has been made globally. In 1990, an estimated 12.6 million deaths occurred among under the age five children and it declined to 5.3 million in 2018. Of the deaths occurred among under the age of five in 2018, 2.5 million (47%) deaths occurred in the first month of life, 1.5 million (29%) deaths occurred at age 1–11 months, and 1.3 million (25%) deaths occurred at age 1–4 years. Sub-Saharan Africa accounts roughly half of all the deaths occurred among under-five children worldwide in 2018. The global under-five mortality rate has dropped by 59%, from 93 deaths per 1000 live births in 1990 to 39 deaths per 1000 live births in 2018. This is equivalent to 1 in 11 children dying before reaching age five in 1990, compared to 1 in 26 in 2018 [2].

There are widespread regional and income disparities in children’s chances of survival with the WHO African region carrying the highest burden of under-five mortality in the world. Sub-Saharan Africa continues to be the region with the highest risk of dying before the age five in the world with 78 deaths per 1000 live births in 2018. This means that 1 child in 13 dying his or her before their fifth birthday, which is 16 times higher than the average ratio of 1 child in 199 in high-income countries and 20 times higher than in the region of Australia and New Zealand [2].

In Ethiopia, according to the recent Ethiopian Demographic and Health Survey (2016 EDHS) report, the under-five mortality rate was 67 deaths per 1000 live births, which means 1 in 15 children in Ethiopia dies before reaching age five. The under-five mortality declined from 166 deaths per 1000 live births in 2000 to 67 deaths per 1000 live births in 2016 in the country, which represents a 60% decrease in under-five mortality over a period of 16 years [3].

Though Ethiopia made an improvement in reduction of under-five mortality in the past two decades, still it is high and therefore more efforts need to be made and appropriate interventions also need to be implemented to meet the Sustainable Development Goals (SDGs) that targeted to reduce under-five mortality rate to at least as low as 25 deaths per 1000 live births by 2030.

In Ethiopia, the under-five mortality rate varies among the geographical regions [4–7]. Southern Nations Nationalities and People’s Region is one of the geographical regions of Ethiopia with high under-five mortality rates [3, 4]. The 2016 EDHS reported that in SNNPR under-five mortality rate was 88 deaths per 1000 live births [3].

Previously conducted studies showed that under-five mortality is determined by various factors including sex of child [8–11], birth order [8, 11–14] size of child at birth [5, 11, 13, 15] breastfeeding status [11, 12, 14–17] preceding birth interval [11–13, 16, 17] type of birth [11, 12, 15–18], place of delivery [8, 11]. Studies also showed that under-five mortality is determined by mother’s age [11] mother age at first birth [4, 5, 10–12, 15] mother’s education [4, 5, 8, 10, 11, 15], sex of the household head [9], household socioeconomic status [4, 16, 17, 19] contraceptive methods use [13, 15], family size [10, 12, 13, 16, 17], types of place of living [11, 13], source of drinking water and toilet facility [9, 12, 16, 17], religion, marital status and employment status of mother [4, 11].

To my knowledge, there is no study that has been done at regional level in SNNPR on determinant factors of Under-five mortality. Identifying the determinant factors that are associated with under-five mortality is important to inform policy makers to provide relevant alternative interventions or strengthen the existing interventions so as to reduce under-five mortality to the level it is expected. Thus, this study aimed to identify determinant factors associated with under-five mortality in SNNPR, Ethiopia.

## Methods

### Data source and study design

The data used for this study were extracted from national cross-sectional demographic and health survey, 2016 EDHS (Ethiopian demographic and health survey) which was conducted from January 18 to June 27, 2016. The 2016 EDHS was designed to provide estimates for the health and demographic variables of interest for Ethiopia as a whole, for urban and rural areas separately, and for each of the nine regions and the two administrative cities of Ethiopia. The 2016 EDHS sample was selected in two stages. In the first stage, a total of 645 enumeration areas were selected with probability proportional to enumeration area size. In the second stage, 28 households per cluster were selected with an equal probability systematic selection from the household list. Among the 2016 EDHS data, the kids data (data on children born in the 5 years prior to the interview) corresponding to SNNPR were used for final analysis.

### Study area

The Southern Nations, Nationalities, and People’s Region (SNNPR) is one of the administrative regions of Ethiopia. The region has estimated population of 19.17 million in 2017.

### Variables of the study

#### Dependent variable

The dependent variable is death of under-five children.

#### Predictor variables

Based on previously conducted studies, various demographic, socio-economic, biological, and environmental factors were included as predictor variables: mother’s age at first birth, mother’s current age, mother’s educational level, sex of household, mother’s work status, contraceptive methods, household wealth index, family size, sex of child, birth order, birth type, size of child at birth, breastfeeding status, place of residence, place of delivery, toilet facility, and source of drinking water.

### Data analysis

Data analysis was done using STATA version 14.

### Statistical analysis

The background characteristics of the sample were summarized using descriptive statistics like frequencies and percentages. Bivariate analysis using Pearson chi-square test was used to test the association between under-five mortality and the predictor variables. The multivariable logistic regression model was applied to identify the determinant factors of under-five children mortality.

## Results

### Descriptive statistics

In this study, a total of 1277 under-five children were included. Of the total under-five children, 71(5.6%) of them were reported dead before the age of 5 years (Table 1).

**Table 1:** Prevalence of under-five mortality in SNNPR, Ethiopia

		Counts	Percent
**Child status**	Died	71	5.6
	Alive	1206	94.4
	Total	1277	100

### Result of bivariate analysis

As it shown in Table 2, the higher percentage (97.2%) of under-five mortality was observed among rural children than the urban children (2.8%). It was also higher among male children (6.8%) than female children (4.2%). Similarly, it was higher among children of multiple births (23.1%) than children of single birth (5.2%). Regarding birth order, it was highest among children whose birth order was sixth or above (6.3%) while it was lowest among children whose birth order was second or third (4.6%). It was also highest among children whose size at birth was smaller (8.5%) and lowest among children whose size at birth was average (3.5%).

**Table 2:** Bivariate analysis of under-five mortality by background characteristics of mothers and children

Characteristics/Variables	Total counts	U5M (Counts)	U5M (Percent)	***P***-value
**Place of residence**				
Rural	1182	69	5.8	0.127
Urban	95	2	2.1	
**Mother’s age at first birth**				
Less than 20	723	38	5.3	0.158
20–29	451	23	5.1	
30 or older	103	10	9.7	
**Mother’s current age**				
15–19	29	2	6.9	0.545
20–24	203	8	3.9	
25–29	418	20	4.8	
30–34	301	18	6.0	
35 or older	326	23	7.1	
**Sex of child**				
Male	657	45	6.8	0.021
Female	620	26	4.2	
**Birth order**				
First	230	12	5.2	0.016
Second or Third	389	18	4.6	
Fourth or Fifth	309	19	6.1	
Sixth or above	349	22	6.3	
**Mother’s education level**				
No education	759	47	6.2	0.420
Primary	426	21	4.9	
Secondary	60	3	5.0	
Higher	32	0	0	
**Contraceptive methods**				
No	757	49	6.5	0.086
Yes	520	22	4.2	
**Household wealth index**				
Poorest	234	13	5.6	0.042
Poorer	306	21	6.9	
Middle	303	16	5.3	
Richer	287	18	6.3	
Richest	147	3	2.0	
**Family size**				
Less than 5	347	28	8.1	0.017
5 or more	930	43	4.6	
**Size of child at birth**				
Larger	439	28	6.4	0.010
Average	567	20	3.5	
Smaller	271	23	8.5	
**Sex of household**				
Male	1107	58	5.2	0.047
Female	170	13	7.6	
**Place of delivery**				
Home	906	58	6.4	0.005
Health Institutions	371	13	3.5	
**Birth type**				
Single	1251	65	5.2	0.000
Multiple	26	6	23.1	
**Breastfeeding status**				
No	415	47	11.3	0.000
Yes	862	24	2.8	
**Mother’s work status**				
Not working	795	33	4.2	0.030
Working	482	38	7.9	
**Toilet Facility**				
No facility	304	31	10.2	0.240
Have facility	973	40	4.1	
**Source of drinking water**				
Unprotected	580	37	6.4	0.500
Protected	697	34	4.9	

The under-five mortality decreases with increased level of education of mothers. It was highest among children whose mothers were not educated (6.2%) while no under-five mortality was observed among children whose mothers attended higher than secondary school. Similarly, it was highest among the children whose mothers aged 30 or older at first birth (9.3%) and lowest among the children whose mothers aged 20 to 29 at first birth (5.1%).

The under-five mortality varied with household wealth index, mother’s work status, family size and sex of head of household. It was highest among households with wealth indices poorer (6.9%) and lowest among households with wealth indices richest (2.0%). Similarly, it was higher among the children whose mothers were working (7.9%) than those whose mothers were not working (4.2%). Regarding family size, the under-five mortality was higher among the children born in families with family size of less than (8.1%) than those born in families with family size of five or more (4.6%). Similarly, it was higher among children who were from households headed by females (7.6%) than among those who were from households headed by males (5.2%).

Under-five mortality also varied with contraceptive methods, place of delivery, breastfeeding and source of drinking water. It was higher among children whose mothers did not use any of the contraceptive methods (6.5%) than those whose mothers used any of the contraceptive methods (4.2%). It was higher among children born at home (6.4%) than those born at health in institutions (3.5%). Similarly, it was higher for the children who were not breastfed (11.3%) than those who were breastfed (2.8%). There was also higher (6.4%) under-five mortality among children who used unprotected source of drinking water than those who used protected source of drinking water (4.9%).

The multivariable logistic regression analysis (Table 3) revealed that sex of a child, birth order, size of a child at birth, place of delivery, birth type, breastfeeding status, and family size were statistically significant determinant factors of under-five children mortality at 5% level of significance. The odds of under-five children death among females was 0.552 (OR = 0.552, 95% CI: (0.327, 0.932)) times lower than males. Children with birth order of second or third (OR = 1.316, 95% CI: (1.097, 2.343)), children with birth order of fourth or fifth (OR = 1.934, 95% CI: (1.678, 3.822)) and children with birth order of sixth or above (OR = 3.980, 95% CI: (2.352, 6.734)) were associated with increased odds of under-five death compared to those with birth order of first. Similarly, children from families with five or more members had higher odds (OR = 3.397, 95% CI: (1.702, 6.782)) of under-five death compared to those from families with less than five members.

**Table 3:** Result of multivariable logistic regression analysis

Variables	OR	***p*** > z	95% CI of OR	
**Sex of child (Ref: Male)**				
Female	0.552	0.026^a^	0.327	0.932
**Birth order (Ref: First)**				
Second or Third	1.316	0.039^a^	1.097	2.343
Fourth or Fifth	1.934	0.018^a^	1.678	3.822
Sixth or above	3.980	0.027^a^	2.352	6.734
**Household wealth index (Ref: Poorest)**				
Poorer	1.125	0.757	0.532	2.379
Middle	0.825	0.639	0.371	1.839
Richer	0.967	0.934	0.440	2.128
Richest	0.330	0.100	0.088	1.235
**Family size (Ref: < 5)**				
5 or more	3.397	0.001^a^	1.702	6.782
**Place of delivery (Ref: Home)**				
Health Institutions	0.449	0.011^a^	0.228	0.681
**Birth type (Ref: Single)**				
Multiple	1.472	0.002^a^	1.289	2.746
**Size of child at birth (Ref: Larger)**				
Average	1.324	0.094	0.700	2.504
Smaller	1.714	0.024^a^	1.120	2.623
**Sex of household head (Ref: Male)**				
Female	0.555	0.160	0.390	1.879
**Breastfeeding status (Ref: No)**				
Yes	0.657	< 0.0001 ^a^	0.393	0.864
**Mother’s work status (Ref: Not working)**				
Working	1.448	0.158	0.866	2.0419

*Ref* Reference category, *CI* confidence Interval, *OR* Odds Ratio, ^a^ Significant at 5% level of significance.

Children with smaller size at birth (OR = 1.714, 95% CI: (1.120, 2.623)) were associated with increased odds of under-five death compared to those with larger size at birth. Similarly, children of multiple births (OR = 1.472, 95% CI: (1.289, 2.746)) were associated with increased odds of under-five death compared to children of single birth.

Children born at health institutions had lower odds (OR = 0.449, 95% CI: (0.228, 0.681)) of under-five death compared to those born at home. Similarly, children who were breastfed, for any period, had lower odds (OR = 0.657, 95% CI: (0.393, 0.864)) of under-five death compared those who were not breastfed.

### Goodness-of-fit

The goodness of fit of the model was examined by using Hosmer-Lemeshow goodness-of-fit test.

Hypothesis test:

H_0_: The model is a good fit Versus.

H_1_: The model is not a good fit.

Since the *p*-value = 0.414 > 0.05, we do not reject the null hypothesis and we conclude that the model is a good fit (Table 4).

**Table 4:** Hosmer-Lemshow goodness-of-fit test

Hosmer-Lemeshow test	*p*-value
8.204	0.414

## Discussion

Of the total of 1277 under-five children included in this study, 71(5.6%) of them were reported dead before the age of 5 years. Majority (92.6%) of the children were living in rural areas while the remaining 7.4% of them were living in urban areas. 51.4% of the children were males and the remaining 48.6% of them were females. Majority (98%) of the children’s birth type was single and only 2% of the children’s birth type was multiple. About two-third (67.5%) of the children were breastfed while the remaining 32.5% were not. More than two-third (70.9%) of the children were born at home and only 29.1% of them were born at health institutions. The majority (30.5%) of the children had birth order of second or third, about 24% of them had birth order of fourth or fifth, 18% of them had birth order of first and the remaining 27.3% of them had birth order of sixth or above. Majority (44.4%) of the children had average size, about 34.4% of them had size of above average while the remaining 21.2% of them had size of below average at birth. More than two-third (72.8%) of the children were from families with family size of five or more while the remaining 27.2% of them were from families with family size of less than five (Table 5).

**Table 5:** Background characteristics of mothers and children in SNNPR Region, Ethiopia

Characteristics/Variables	Counts	Percent
**Place of residence**		
Rural	1182	92.6
Urban	95	7.4
**Mother’s age at first birth**		
Less than 20	723	56.6
20–29	451	35.3
30 or older	103	8.1
**Mother’s current age**		
15–19	29	2.3
20–24	203	15.9
25–29	418	32.7
30–34	301	23.6
35 or older	326	25.5
**Sex of child**		
Male	657	51.4
Female	620	48.6
**Birth order**		
First	230	18.0
Second or Third	389	30.5
Fourth or Fifth	309	24.2
Sixth or above	349	27.3
**Mother’s education level**		
No education	759	59.4
Primary	426	33.4
Secondary	60	4.7
Higher	32	2.5
**Contraceptive methods**		
No	757	59.3
Yes	520	40.7
**Household wealth index**		
Poorest	234	18.3
Poorer	306	24.0
Middle	303	23.7
Richer	287	22.5
Richest	147	11.5
**Family size**		
Less than 5	347	27.2
5 or more	930	72.8
**Size of child at birth**		
Larger	439	34.4
Average	567	44.4
Smaller	271	21.2
**Sex of household**		
Male	1107	86.7
Female	170	13.3
**Place of delivery**		
Home	906	70.9
Health Institutions	371	29.1
**Birth type**		
Single	1251	98.0
Multiple	26	2.0
**Breastfeeding status**		
No	415	32.5
Yes	862	67.5
**Mother’s work status**		
Not working	795	62.3
Working	482	37.7
**Toilet Facility**		
No facility	304	23.8
Have facility	973	76.2
**Source of drinking water**		
Unprotected	580	45.4
Protected	697	54.6

The result of the multivariate logistic regression analysis revealed that male children were at higher risk of under-five mortality compared to female children. This result is consistent with the result of previous studies [9, 11, 20–22]. The possible explanation for this might be that male children are biologically weaker than their female counterparts due to a fundamental genetic advantage.

Children of higher birth order had higher risk of under-five mortality compared to those children of lower birth order. This is consistent with the previous study [13, 16]. The reason for this might be that as birth order increases care given to child by mother decreases because of having more children.

It was found that children from larger family size were more likely to die before the age of 5 years compared to those children from smaller family size. This is consistent with findings of the previously done studies [13, 17]. This might be due to that as family size increases, the share of foods and other limited sources required for the child survival decreases.

Children born at home were more likely to die before the age of 5 years compared to those born at health institutions. This result agrees with the finding of the previous study [8, 16]. This might be due to the fact that children born at home are more susceptible to infections.

It was also found that children who were breastfed were associated with lower odds of under-five mortality compared to those who were not breastfed. This result is consistent with the results of previous studies [10, 14, 17]. The possible explanation for this might be that breast milk helps build and support baby’s immune system that defends body against invaders, such as viruses and bacteria.

It was also found that children of multiple births were at higher risk of under-five mortality compared to singletons. This is consistent with findings of the previously studies [5, 17, 18, 20]. The possible explanation for this might be that foods and other limited resources and care given to child by mother are shared.

Children with smaller size at birth were found to have higher odds of under-five mortality than those with larger size at birth. This result agrees with the result of previous study [16].

## Conclusions

The study aimed at identifying the determinants of under-five mortality in Southern Nations Nationalities and People Region, Ethiopia. A total of 1277 under- five children were included in the study. It was found that sex of a child, birth order, size of a child at birth, place of delivery, birth type, breastfeeding status, and family size were significant determinants of under-five mortality in SNNPR, Ethiopia.

## Limitations of the study

One of the limitations of this study was that factors such as preceding birth interval, number of antenatal care visits during pregnancy, which are supposed to be associated with under-five mortality were not included in the study because of high missing values in the data. The other limitation was that mothers might forget events that occurred in the past 5 years preceding the survey which could have introduced recall bias.

## Data Availability

The data used for this study for final analysis are available from the author upon reasonable request.

## Abbreviations

CSA: Central Statistics Agency
DHS: Demographic and Health Survey
EDHS: Ethiopian Demographic and Health Survey
SDGs: Sustainable Development Goals
OR: Odds Ratio
SNNPR: Southern Nations, Nationalities, and Peoples’ Region
U5M: Under-five mortality
WHO: World Health Organization
